# The application of genetics approaches to the study of exceptional longevity in humans: potential and limitations

**DOI:** 10.1186/1742-4933-9-7

**Published:** 2012-04-23

**Authors:** Anna Ferrario, Francesco Villa, Alberto Malovini, Fiorella Araniti, Annibale A Puca

**Affiliations:** 1 IRCCS Multimedica, Via Fantoli 16/15, 20138, Milan, Italy; 2 University of Pavia, Via Ferrata 1, 27100, Pavia, Italy; 3 Università degli Studi di Salerno, Via S. Allende, 84081, Baronissi, Italy

**Keywords:** Aging, Centenarians, Longevity

## Abstract

The average life-span of the population of industrialized countries has improved enormously over the last decades. Despite evidence pointing to the role of food intake in modulating life-span, exceptional longevity is still considered primarily an inheritable trait, as pointed out by the description of families with centenarian clusters and by the elevated relative probability of siblings of centenarians to become centenarians themselves. However, rather than being two separate concepts, the genetic origin of exceptional longevity and the more recently observed environment-driven increase in the average age of the population could possibly be explained by the same genetic variants and environmentally modulated mechanisms (caloric restriction, specific nutrients). In support of this hypothesis, polymorphisms selected for in the centenarian population as a consequence of demographic pressure have been found to modulate cellular signals controlled also by caloric restriction. Here, we give an overview of the recent findings in the field of the genetics of human exceptional longevity, of how some of the identified polymorphisms modulate signals also influenced by food intake and caloric restriction, of what in our view have been the limitations of the approaches used over the past years to study genetics (sib-pair-, candidate gene association-, and genome-wide association-studies), and briefly of the limitations and the potential of the new, high-throughput, next-generation sequencing techniques applied to exceptional longevity.

## Mechanisms of longevity

Life expectancy in the US 1900 Birth Cohort Study was found to be 51.5 years for males and 58.3 years for females, and currently 1/10,000 individuals reach 100 years of age: this prevalence is quickly changing and will probably soon approach 1/5,000 [1]. The increased ability to reach 100 years old in industrialized countries over the last 160 years most likely reflects a rise in life expectancy — quantified as 3 months/year for females — as a consequence of improvements in diet and a reduced exposure to infection and inflammation [2]. In favour of diet as a modulator of longevity, the Elderly Prospective Cohort Study (EPIC) identified a reduced overall mortality among the elderly consuming a modified Mediterranean diet in which saturated fatty acids were substituted for monounsaturated ones [3].

Centenarians, despite being exposed to the same environmental conditions as members of the average population, manage to live much longer; moreover, as a consequence of demographic selection, centenarians have a compression of morbidity and mortality towards the end of their life-span [4]. Genetically, this compression in morbidity and mortality is correlated with the enrichment of protective alleles and the depletion of detrimental ones. These alleles run in families, as shown by the familiar clustering of exceptional longevity. It has been estimated that genetic variants account for at least 25% of human life-span, and for even a larger proportion in individuals living to extreme age [5,6].

The potential overlap of hits for environmentally and genetically mediated predisposition for extreme longevity in centenarians is highlighted by the association of genetic variants of genes that regulate, or that are regulated by, nutrient metabolism, such as apolipoprotein E (APOE) and Forkhead box O3A (FOXO3A) [7]. Inheritance of the longevity phenotype is underlined by the low all-cause and cardiovascular-disease mortality rates observed in offspring of centenarians when compared with an age-matched population [8]. The study of centenarian offspring has revealed biomarkers of longevity, like low serum levels of heat shock proteins (HSP), large lipid particle sizes, and high membrane palmitoleic acid paired with a low peroxidation index [9-11]. In addition, centenarians have high glucose tolerance and insulin action, and low heart-rate variability (HRV), which contrasts with the decline observed in control populations [12,13].

Genetic variants that modulate human longevity should also modulate cellular pathways that control key aspects of the aging process, such as oxidative stress-induced apoptosis (RAS/ERK pathway), DNA repair (NF-KB1 and hTERT), senescence (p53), mitochondrial biogenesis (AMPK) and cell survival (PI3K/AKT pathway). Many of these pathways cross-talk with each other and are finely regulated in order to obtain the best trade-off between advantages and disadvantages of inducing either cell survival or apoptosis/senescence. The best trade-off is mostly tissue specific and can change with the degree of the tissue’s differentiation (stem versus differentiated cells) and health status (healthy, proliferative, degenerative or ischemic). It is plausible that genetic variants that impact on human longevity affect genes that are expressed selectively in some tissues and/or in specific stages of differentiation. Although aging has been considered an evolutionary adaptation for fighting cancer through the activation of processes like senescence, there are signals that are able to activate cell-specific responses, e.g. induction of apoptosis by adenosine monophosphate-activated protein kinase (AMPK) in cancer cells, while inducing survival in healthy cells. AMPK recognizes high levels of AMP, and is activated by caloric restriction, physical exercise, metformin, essential amino acids and alpha-lipoic acid [14]. Furthermore, AMPK induces mitochondrial biogenesis, autophagy and beta-oxidation of free fatty acids [15-17]. Reduction of fatty acid beta-oxidation promotes diabetes, obesity and, ultimately, aging.

How can these cellular signals be altered without producing side effects? The answer could be found in the centenarian genome through the identification of genetic variants that have been selected or dropped for their role in human health. AMPK signalling is an example of how variation in the quality and the amount of food could impact on longevity by modulating signals that are influenced by genetic variants selected in centenarians. To be noted, Sirtuin 1 (SIRT1), despite its role in many cellular processes, induces survival and is not affected by polymorphisms that associate with exceptional longevity in humans, perhaps on account of its critical role in tumours as well [18].

## The candidate gene approach and associations with longevity

To date, only a few genetic variants have been consistently found associated with exceptional longevity in humans (Table 1). The most convincing result discovered to date by a candidate gene approach is the reduction of the APOE ϵ4 allele in centenarians as a result of its correlation with cardiovascular disease and Alzheimer’s disease [7]. APOE knockout mice develop atherosclerosis, so genes that modulate vascular integrity are potential candidates that may be identified in genetic association studies of exceptional longevity [19].

**Table 1:** Genes and variants found correlated with longevity in humans

**Gene**	**Variant**	**Occurrence in centenarians**	**Previous disease correlations**	**Potential role in longevity**	**Ref.**
APOE	ε4*	reduced	Alzheimer’s and cardiovascular diseases	Maintenance of vascular integrity	[7]
FOXO3A	rs2802292*	more present	none	Control of cell homeostasis	[20]
CAMK4	rs10491334^†^	more present	hypertension	Modulation of CREB, SIRT and FOXO3A	[21]
ATXN1	rs697739	more present	amyotrophic lateral sclerosis (age of onset)	Modulation of CREB	[21]
DCAMKL1	rs9315385	more present	heart rate variability	Modulation of CREB	[21]

*, result obtained by a candidate gene approach and replicated in other studies; ^†^, result obtained from a GWAS and confirmed in a replication cohort.

The FOXO3A rs2802292 allele is another variant found associated with exceptional longevity across populations [20,22-24]. Nevertheless, this polymorphism has no apparent impact on the functions of FOXO3A and it is not in LD with functional variants. However, FOXO3A is part of a pathway associated with longevity: IGF1/PI3K/PDK1/AKT/FOXO. Animal studies from worms to mice have shown that genetic modifications are able to postpone aging by modulating the IGF1/PI3K/PDK1/AKT/FOXO pathway [25]. This pathway regulates many aspects of cell homeostasis, from cell survival and proliferation to oxidative stress response, depending on concomitant stimuli [26,27]. Interestingly, individuals with short stature due to a lack of growth hormone, which is upstream of the IGF1/PI3K/PDK1/AKT/FOXO pathway, have a reduced incidence of tumours and diabetes [28]. The IGF1/PI3K/PDK1/AKT/FOXO pathway is strongly modulated by caloric restriction, as are the levels of AMPK and SIRT1. Glucose and insulin resistance also modulates this pathway, which associates with inefficient uptake of glucose, inducing its overstimulation and aging. To some extent, genetic alterations of the insulin-like growth factor 1 (IGF1) receptor that alter the IGF signalling pathway confer an increase in propensity for longevity [29].

Adenosine deaminase, RNA-specific (ADAR) and telomerase gene variants have also been associated with human longevity [30,31]. However, with the exception of APOE and FOXO3A variants, none of the many candidate genetic variants tested to date have been consistently replicated across populations. This is possibly on account of differing environmental stimuli generating inconsistent demographic pressures, making results, as a consequence, irreproducible [32].

**Figure 1:**
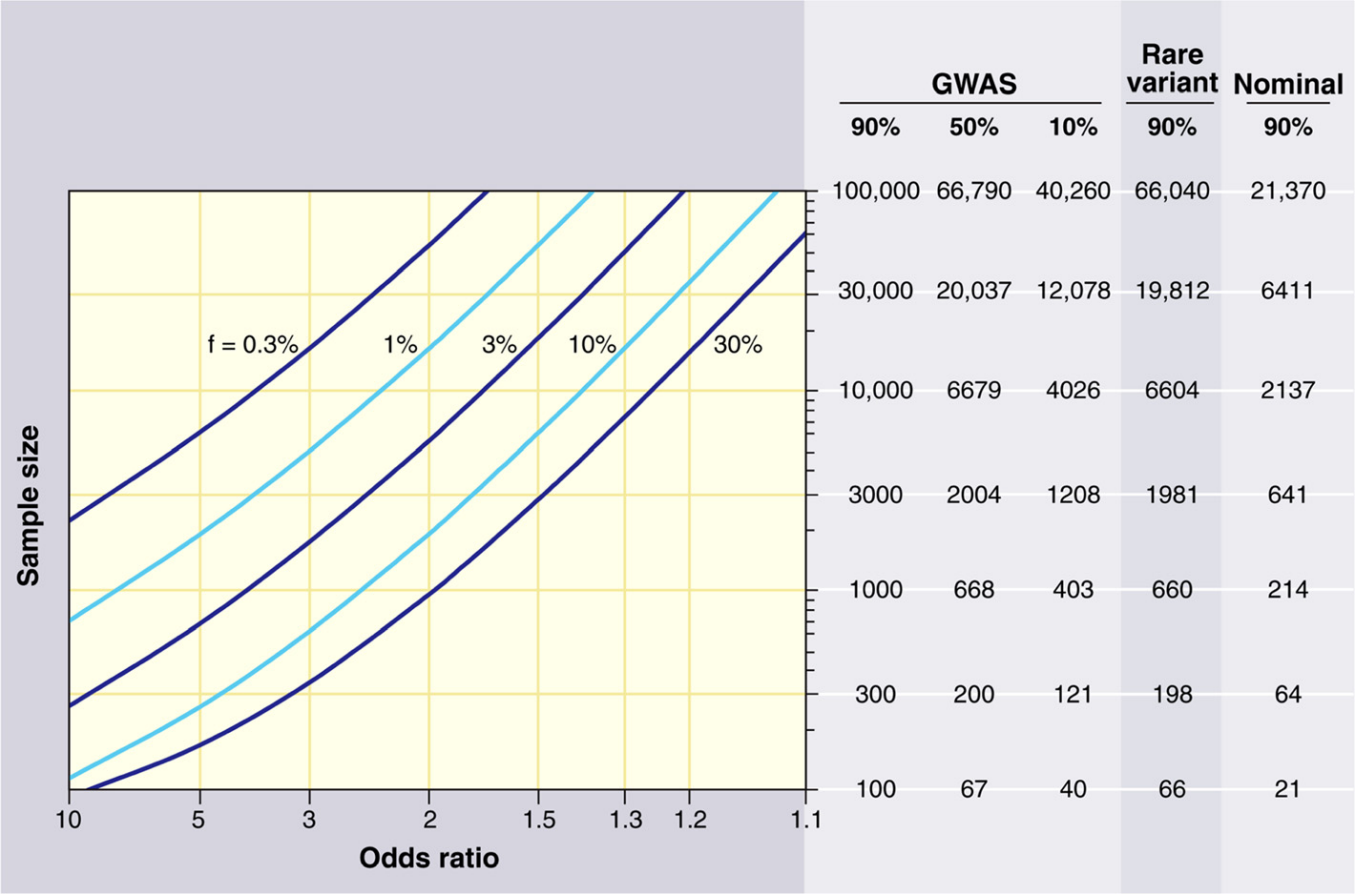
**Sample sizes required for genetic association studies.** The graph shows the total number N of samples (consisting of N/2 cases and N/2 controls) required to map a genetic variant as a function of the increased risk due to the disease-causing allele (x axis) and the frequency of the disease-causing allele (various curves). The required sample size is shown in the table on the right for various different kinds of association studies [33]. Reproduced with permission from The American Association for the Advancement of Science.

Other potential problems that generate false positive and false negative results include the low power of studies using small sample sizes, and a lack of a suitable control for the genetic admixture. On the first point, an extremely instructive review has been written by Altshuler, Daly and Lender, who calculate the power of a study based on the number of individuals genotyped, the number of tested hypotheses, and the frequency of the allele tested for a specific OR [33]. From the graph given in their review (Figure 1), it is clear that for the OR expected in human exceptional longevity (between 1.2 and 2), the power of a study is highly dependent upon the number of hypotheses tested. If we consider that many laboratories test their genetic variants and publish only the positive results they find, the few hundred individuals typically used in a candidate gene approach on exceptional longevity are not sufficient to minimize false-positive findings.

With regard to the appropriate control for a given genetic admixture, it is possible to correct for systematic ancestry differences between cases and controls — an effect that can cause false associations — by the application of principal components analysis to the genotyping of thousands of SNPs with chips [34].

Finally, because the candidate gene approach is hypothesis driven, the functional validation of the coded protein in signals important for aging and longevity do not add strength to the finding, while as we will see, this is an opportunity for hypothesis-free approaches, such as the genome-wide association study (GWAS) and sib-pair analysis.

## Genome-wide association studies and exceptional longevity

GWASs are hypothesis-free efforts that generate findings that need to be replicated in independent populations. In the case of exceptional longevity, success in replicating initial findings is negatively influenced by the differences in participant ages, gender and disease status distribution across the analysed populations. Furthermore, the GWAS approach suffers from the multiple-testing statistical penalty that forces the adoption of very low p values of significance, hence favouring the phenomenon of the winning course, i.e. the enrichment of false-positive associations among the dozens of top findings [35]. For these reasons, recent GWASs have failed to find variants that cross-validate across populations — with the exception of the known APOE locus — pointing to the need of much larger studies or alternative study designs in order to discover common polymorphisms with smaller genetic effects and rare variants with high penetrance that influence exceptional longevity [36,37].

Regarding the power of capturing true associations in GWAS efforts, the Altshuler, Lander and Daly review calculation clearly shows that a sample size comprising thousands of individuals is needed to identify the expected OR in a GWAS on exceptional longevity [33]. Thus, a GWAS on exceptional longevity can be considered only a hypothesis-generating effort to be used in conjunction with other studies.

For the above reasons, in our recently published GWAS on individuals enrolled in the Southern Italian Centenarian Study (SICS), we not only attempted to progressively reduce the number of tested hypotheses, but also considering the redundancy (non-independence) of the many SNPs represented on the Illumina 317 k bead-chip employed, we decided to use a 300 k SNP screening of the SICS individuals as a hypothesis-generating set, adopting a Genomic Control (GC)-corrected p-value < 1e-4 threshold (which is a less stringent threshold than *p* < 5x10-2/317000 = 1.5x10-7) for the replication, evaluating allelic, genotypic, dominant and recessive genetic association models [21].

Initial screening of SICS individuals identified CAMK4 rs10491334, a variant that had been already established among the top 5 SNPs in the Framingham Heart Study on diastolic high blood pressure [38]. The fact that CAMK4 rs10491334 associates also with hypertension is reassuring in that hypertension and longevity are regulated by common pathways. In fact, mice with genetic ablation of the angiotensin II type1 receptor — the key regulator of blood pressure — had increased expression of the longevity gene Sirt3 and improved survival [39]. Interestingly, rs10491334 correlated with CAMK4 protein expression, and functional studies revealed the ability of CAMK4 protein to modulate SIRT1 and FOXO3A.

The ataxin-1 (ATXN1) rs697739 allele was another variant found among the top findings of our GWAS on SICS individuals. This polymorphism had been previously associated with the age of onset of sporadic amyotrophic lateral sclerosis, a disease of unknown cause characterized by slowly progressive degeneration of motor neurons and that usually occurs in patients aged 40–60 years [40]. ATXN1 is the gene responsible for spinocerebellar ataxia type 1 and antagonizes the neuronal survival function of myocyte enhancer factor-2 (MEF2) [41].

MEF2 transcription repression by cabin1-HDAC4 is removed by CAMKIV activation, and this suggests that MEF2 is a common downstream target of CAMKIV and ATXN1 [42,43].

In addition to CAMKIV rs10491334 and ATXN1 rs697739, the rs9315385 allele of doublecortin and Ca^2+^/calmodulin-dependent kinase-like-1 (DCAMKL1) was a third top finding of our study. DCAMKL1 has structural similarity with CAMKIV, but despite this, it represses CAMKIV-induced activation of cAMP response element-binding (CREB) protein via phosphorylation of transducer of regulated CREB activity 2 (TORC2) at Ser171 [44]. DCAMKL1 rs9315385 was previously associated with total power of HRV [45]. A reduced HRV is a marker of autonomic dysfunction and is associated with an increased risk of cardiovascular morbidity and mortality [46]. HRV-parasympathetic function decreases up to the eighth decade of life, followed by an increase to higher levels — similar to those found in a younger population — in nonagenarians and centenarians [13]. Similarly to CAMKIV, DCAMKL1 and ATXN1 are expressed mainly in brain. These data support the importance of the CAMKIV/CREB pathway in regulating the aging process.

A brief mention needs to be made here on the cutting-edge, genetic signature paper by Sebastiani et al. that very elegantly proved that a complex analysis on 281 SNPs allowed to define clusters of individuals that aged differently based on their genetic signature [47].

## Linkage analysis and exome sequencing

Sib-pair analysis has been for a while the only tool available for the identification of chromosomal regions that potentially harbour genetic variants influencing the phenotype of interest. The approach can identify excess allele sharing, and was initially performed with microsatellites. It consists in the identical-by-descendant analysis of very informative markers that reconstruct the haplotype of parents and how they co-segregate in their offspring. We performed such an analysis on a unique collection of sib-pairs and their families, collected by the New England Centenarian Study (NECS), and identified a significant peak on 4q25 [48]. Follow-up analysis failed to identify genetic variants that could explain the initial linkage finding. Rare mutations that segregate in centenarian siblings are eventually captured by sibling-pair analysis, but this cannot be the case for genetic association studies that loose power as the allele frequency of the tested polymorphisms drops. Furthermore, it is possible that linkage efforts identify chromosomal regions where more causative genetic variants reside, and thus the sum of their effects determines the linkage result, whereas with follow-up genetic association approaches, the analysis involves one common polymorphism at a time or, eventually, haplotypes. Attempts to replicate the initial linkage did not succeed, except for an initial replication effort that successfully replicated the linkage at D4S1564 [49,50]. A negative replication effort can be due to an initial false-positive finding or to the diversity of the populations used for the replication effort, in terms of genetic background, the environment applying the demographic pressure, the ages of the participants, the number of families and the genetic markers adopted. Recently, Kunkel’s laboratory published a well-performed re-analysis of a part of the sib-pairs used in the initial study, plus new sib-pairs recruited by Elixir Pharmaceuticals [51]. To be noted, some of the largest and more impressive families — those that were genotyped upfront and that showed immediately a significant linkage on 4q25 in the original study — were either not analysed or done so only in part by this second effort. The new analysis adopted a high-density marker panel of SNPs to genotype the patients, allowing a better coverage of the genome. They did not replicate the chromosome 4q25 finding, except when the same stringent criteria were adopted to select a sub-set of centenarian families. Interestingly, a new peak on chromosome 3p24-22 reached significant threshold, and a second peak was highly suggestive of linkage at 9q31-34. This latter peak appeared also in the previous analysis with microsatellites, even if less robustly. The attempt to identify the genetic variant/variants responsible for the 4q25 peak pointed to the initial encouraging genetic variant in the promoter of the microsomal triglyceride transfer protein (MTP) gene [52]. Unfortunately, the finding was not replicated by an independent effort and by our analysis that included more controls [32,53].

It is plausible that different approaches are needed to follow up genetic linkage findings, to point to the identification of rare variants that co-segregate in families. To this end, exome sequencing data, intersected with linkage data, could give rise to interesting results.

To be noted, the 4q25 locus harbours elongation of very long chain fatty acids protein 6 (ELOVL6), the elongase that transforms C16:0 into C18:0 and C16:1 into C18:1. Polymorphisms in this gene have been associated with insulin sensitivity; a mouse deficient for this gene carried high doses of C16:1 (palmitoleic acid) and did not acquire insulin resistance after a high-fat diet [54,55]. C16:1 has been identified as an adipose tissue-derived lipid hormone that strongly stimulates muscle insulin action and suppresses hepatosteatosis [56]. Genetically modified, long-living worms have an incredible correlation between their increase in life-span and their palmitoleic acids levels [57]. This is stunning if we consider the increased level of palmitoleic acid that we observed in centenarians’ offspring and that the gene of the major modifier of palmitoleic acid levels (i.e. ELOVL6) is located in the 4q25 longevity locus [11,48]. The re-sequencing in centenarians of this gene could bring to the identification of rare variants able to influence its activity.

Thus, the old approach of linkage analysis when combined with the new technologies of high massive re- sequencing could produce novel and interpretable results. Re-sequencing alone, because of the enormous amount of information generated, would force the application of a huge statistical correction for the multiple testing, which would cause the loss of most, if not all, the potential findings, as happens with GWAS.

Furthermore, multivariate models, based on machine-learning algorithms (i.e. Bayesian networks [58], classification and regression trees — CART [59] — and support vector machines — SVM [60]), are able to overcome the limitations of the usual “*one-SNP-at-the-time*” testing strategies usually employed for identifying causative variants. In particular, these kinds of approaches allow for a more in-depth comprehension of the molecular mechanisms underlying multifactorial traits, such as longevity, which result from the interaction of genetic variants (SNPs, mutations) and environmental and clinical determinants (e.g. diet, stress, comorbidities). In this context, bioinformatics plays a key role, allowing genetic information to be managed at a genome-wide level and to be integrated with the clinical information available.

## Concluding remarks

Despite the enormous progress achieved by DNA- investigating technologies, such as SNP arrays and exome capturing/re-sequencing, the current knowledge on how genetic variants influence exceptional longevity in humans is still based on the old candidate gene approaches. The adoption of innovative study designs combined with novel genetic platforms and innovative statistical methods hopefully will bring to the identification of new intervention points at which to modulate aging and the diseases of aging.

## Abbreviations

ADAR, adenosine deaminase RNA-specific; AMPK, Adenosine monophosphate-activated protein kinase; APOE, apolipoprotein E; ATXN1, Ataxin 1; CAMK, Ca2+/calmodulin-dependent protein kinase; CART, classification and regression tree; CREB, cAMP response element-binding; DCAMKL1, doublecortin and Ca2+/calmodulin-dependent kinase-like-1; ELOVL6, elongation of very long chain fatty acids protein 6; EPIC, Elderly Prospective Cohort Study; FOXO, forkhead box O; GC, genomic control; GWAS, genome-wide association study; HRV, heart-rate variability; HSP, heat shock protein; IGF, insulin-like growth factor; LD, linkage disequilibrium; MEF2, myocyte enhancer factor-2; MTP, microsomal triglyceride transfer protein; NECS, New England Centenarian Study; OR, odds ratio; SICS, Southern Italian Centenarian Study; SIRT1, Sirtuin 1; SNP, single nucleotide polymorphism; SVM, support vector machine; TORC2, transducer of regulated CREB activity 2.

## Competing interests

The authors declare that they have no competing interests.

## Authors’ contributions

AAP wrote the first draft; Subsequent drafts were written by AF, FV and FA, who had the overall supervision of the review processing; all authors edited the paper and approved its final version.
